# Effectiveness of low-dose SGLT2 inhibitors in diabetic patients with heart failure: a nationwide cohort study

**DOI:** 10.3389/fcvm.2026.1859003

**Published:** 2026-06-22

**Authors:** Minkwan Kim, Seok-Jae Heo, SungA Bae, In Hyun Jung, Deok-Kyu Cho

**Affiliations:** 1 Division of Cardiology, Department of Internal Medicine, Yongin Severance Hospital, Yonsei University College of Medicine, Yongin, Gyeonggi-do, Republic of Korea; 2 Department of Biomedical Systems Informatics, Yonsei University College of Medicine, Seoul, Republic of Korea

**Keywords:** cohort studies, dose-response relationship, heart failure, prognosis, sodium-glucose co-transporter 2 inhibitors

## Abstract

**Background and objectives:**

SGLT2 inhibitors (SGLT2i) are a cornerstone of guideline-directed therapy for heart failure (HF). However, the optimal dose for HF remains unclear, as no phase II trials have evaluated dose-response in this population. This study aimed to evaluate whether low-dose (5 mg) of SGLT2i provide comparable efficacy and safety compared with the standard 10 mg dose and to non-users.

**Methods:**

This nationwide retrospective cohort study utilized Korean National Health Insurance Service claims data (2013–2018), including 551,760 patients with HF and diabetes, of whom 55,694 were SGLT2i users. After exclusions, propensity-score matching was performed in a 5:5:1 ratio (non-users:10 mg:5 mg). The composite primary outcome was cardiac death, HF hospitalization, or urgent HF-related emergency department (ED) visit. Safety outcomes included ketoacidosis, acute kidney injury (AKI), urinary tract infection, and fractures.

**Results:**

In the matched cohort (2,637 on 5 mg; 13,185 on 10 mg; 13,185 non-users), the 5 mg group was associated with lower risk of the primary outcome (HR: 0.73, 95% CI: 0.66–0.81; *P* < 0.001) and all-cause death (HR 0.85, 95% CI 0.74–0.97; *P* = 0.019) compared with 10 mg, with similar safety profiles. Significant interactions were observed: the relative effect of 5 mg vs. 10 mg differed by sex and body weight for urgent ED visits, and by BMI for AKI. Sensitivity analyses across multiple time points showed consistent results, with the 5 mg group associated with comparable or lower risk compared with the 10 mg group.

**Conclusions:**

In patients with diabetes and HF, 5 mg daily SGLT2i was associated with a comparable risk to 10 mg, warranting randomized dose-finding trials.

## Introduction

Sodium-glucose co-transporter-2 inhibitors (SGLT2i) were initially developed for the treatment of diabetes. However, they have since been widely adopted in clinical practice due to their proven benefits in delaying disease progression and improving outcomes in patients with heart failure (HF) and chronic kidney disease (1–4). Furthermore, comparable cardiovascular benefits have been observed in patients without diabetes, indicating that the positive effects of SGLT2i may stem from mechanisms independent of glucose reduction (1, 5). Until recently, no pharmacological agents had demonstrated efficacy in improving cardiovascular outcomes in patients with HF with preserved ejection fraction, rendering treatment challenging. However, recent evidence supports the clinical utility of SGLT2i in this population. This has led to a paradigm shift, whereby SGLT2i are now recognized not only antidiabetic drugs but also as HF treatments (5, 6). The mechanisms underlying the beneficial effects of SGLT2i in patients with HF have been investigated (7–9). The cardioprotective effects of SGLT2i underlying the reduction in HF hospitalization are mediated by complementary mechanisms, including suppression of the renin-angiotensin-aldosterone system with attenuation of cardiac fibrosis, improved myocardial energetics via ketone oxidation, and hemodynamic unloading through natriuresis and osmotic diuresis (10).

Unlike other HF medications, SGLT2i were initially developed as antidiabetic agents; therefore, no phase 2 trials have been conducted to determine the optimal dosage for HF treatment. Current clinical guidelines recommend a once-daily dose of 10 mg for the treatment of HF (11, 12). Randomized controlled trials have reported that this dose does not significantly increase the risk of adverse effects compared with the control groups (1, 3–6, 13). However, in real-world clinical practice, adverse effects of SGLT2i have led to reduced adherence among some patients. A recent study involving more than 100,000 participants found that 40% of patients discontinued SGLT2i within 1 year (14). Risk factors associated with SGLT2i discontinuation included older age, concurrent diuretic use, and lower body weight (15, 16). To address this issue, this study was designed to evaluate the clinical utility of a low-dose (5 mg) SGLT2i in a large-scale nationwide cohort. As the present study was conducted using claims data and, due to reimbursement restrictions during the study period, only patients with both diabetes and HF could be included in the cohort. Accordingly, we evaluated the efficacy and safety of low-dose SGLT2i specifically in this population with diabetic HF.

## Methods

### Data source and study population

**Figure 1:**
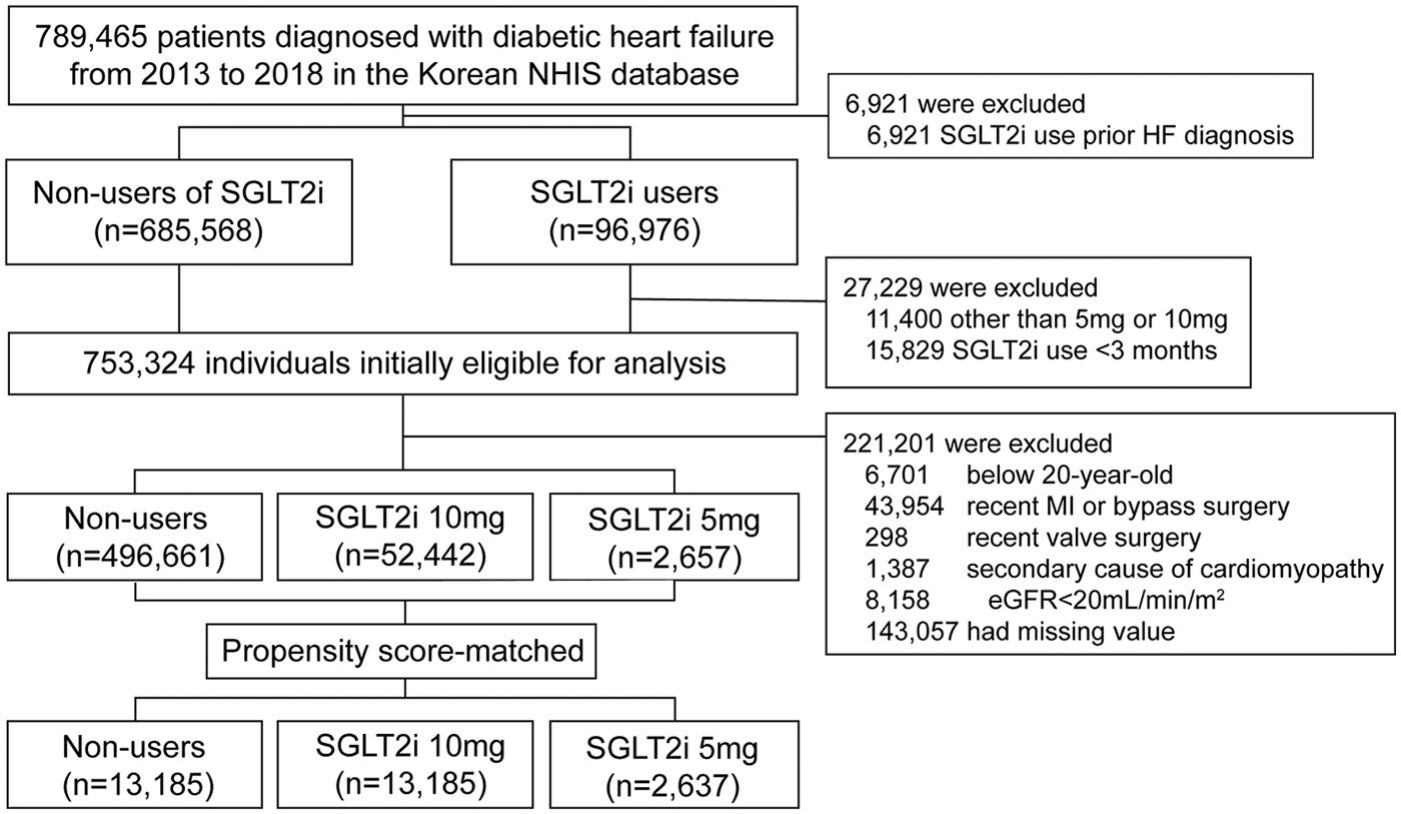
Flow chart of study participant selection and matching process. Among 789,465 patients diagnosed with diabetic heart failure from 2013 to 2018 in the Korean NHIS database, 6,921 patients were excluded because of SGLT2i use before the diagnosis of heart failure. Of the remaining patients, 685,568 were non-users of SGLT2i and 96,976 were SGLT2i users. After excluding 27,229 SGLT2i users who received doses other than 5 mg or 10 mg or used SGLT2i for less than 3 months, 753,324 individuals were initially eligible for analysis. An additional 221,201 patients were excluded because of age below 20 years, recent myocardial infarction or bypass surgery, recent valve surgery, secondary cardiomyopathy, eGFR <20 mL/min/1.73 m², or missing values. Finally, 551,760 patients were included in the analysis: non-users, *n* = 496,661; SGLT2i 10 mg, *n* = 52,442; and SGLT2i 5 mg, *n* = 2,657. Propensity score matching was performed to generate three balanced groups consisting of non-users (*n* = 13,185), SGLT2i 10 mg users (*n* = 13,185), and SGLT2i 5 mg users (*n* = 2,637).

This nationwide cohort study utilized data from the National Health Insurance Service (NHIS) claims database in the Republic of Korea. The NHIS, a government-supervised mandatory health insurance system established in 1989, provides coverage for nearly 97% of the Korean population (17). This extensive database maintains a wide range of health-related information on insured individuals, including demographic characteristics, lifestyle factors, diagnostic records, prescription histories, details of medical procedures and surgeries, and patterns of healthcare utilization, such as hospital visits and admissions (17). The NHIS database was used to identify adults with both HF and diabetes mellitus between January 1, 2013, and December 31, 2018 (initial cohort, *n* = 789,465). Exclusion criteria were: SGLT2i use prior to HF diagnosis (*n* = 6,921); age <20 years (*n* = 6,701); SGLT2i use for <3 months (*n* = 15,829); doses other than 5 mg or 10 mg (e.g., 25 mg; *n* = 11,400); myocardial infarction or coronary artery bypass graft surgery within 90 days of the index prescription (*n* = 43,954); valve surgery during the same period (*n* = 298); secondary cardiomyopathy (cardiac amyloidosis, Fabry disease, obstructive hypertrophic cardiomyopathy, or stress-induced cardiomyopathy; *n* = 1,387); and estimated glomerular filtration rate (eGFR) < 20 mL/min/1.73m^2^ (*n* = 8,158). After exclusions, 551,760 individuals remained for the final analysis (Figure 1). Patients prescribed a once-daily SGLT2i regimen with a single-dose quantity of 0.5 were classified into the 5 mg treatment group.

This study was conducted in accordance with the ethical principles set forth in the 2013 Declaration of Helsinki. Ethical approval was obtained from the Institutional Review Board of Yonsei University College of Medicine, Yongin Severance Hospital (9-2025-0051). The requirement for informed consent was waived owing to the retrospective nature of the study.

### SGLT2i formulations and dose classification

During the study period (2013–2018), neither dapagliflozin nor empagliflozin was marketed in a 5 mg tablet formulation in Korea; the 10 mg tablet was the only commercially available formulation for both agents. The 5 mg dose was therefore achieved through half-tablet administration of the 10 mg tablet, identified in the NHIS prescription records as a single-dose quantity of 0.5. Patients with this prescription pattern were classified into the 5 mg treatment group, and patients with a single-dose quantity of 1 (one full 10 mg tablet) were classified into the 10 mg treatment group, regardless of agent. Other SGLT2i (ipragliflozin, canagliflozin, ertugliflozin) were not included in this study.

### Covariates

This study defined covariates using the same approach as previous studies that utilized the NHIS database (15, 18). Information on age and sex was obtained based on resident identification numbers. Comorbidities such as hypertension, dyslipidemia, ischemic heart disease, atrial fibrillation, prior stroke, and chronic kidney disease were defined according to the criteria outlined in Supplementary Table S1. The Charlson Comorbidity Index (CCI) was calculated as previously described to assess the comorbidity burden for each patient (19). Medications prescribed, including renin-angiotensin-aldosterone system blockers [such as angiotensin receptor–neprilysin inhibitors (ARNI)], beta-blockers, calcium channel blockers, statins, spironolactone, and antiplatelet agents, were identified through prescription records in the database. Physical measurements (body mass index and body weight), lifestyle factors (alcohol consumption and smoking status), and laboratory variables, including hemoglobin (g/dL), fasting glucose (mg/dL), and eGFR (mL/min/1.73m^2^), were obtained from the National Health Screening Program database. For each patient, values measured closest to the index date within the preceding 2 years were used. The eGFR was calculated using the equation established by the Chronic Kidney Disease Epidemiology Collaboration (20).

### Study outcome

The primary outcome was a composite of (1) cardiac death, (2) first HF hospitalization, and (3) an urgent visit to the emergency department (ED). Additionally, individual events and all-cause mortality were also assessed across the three study groups (SGLT2i 5 mg, SGLT2i 10 mg, and control). The index date was defined as the date of first SGLT2i prescription following the diagnosis of heart failure for patients receiving SGLT2i therapy. For patients in the control group who did not receive SGLT2i, the index date was defined as the date of heart failure diagnosis. Patients were followed from the index date until the occurrence of any endpoint component or December 31, 2022, whichever occurred first. For the primary composite analysis, we used a time-to-first-event approach: each patient was followed until the earliest occurrence of any of the three component events, after which the patient was no longer at risk for further composite events. In addition, each individual component (cardiac death, HF hospitalization, urgent ED visit) and all-cause mortality were analyzed separately, with the event of interest counted whenever it occurred during follow-up, regardless of whether any other event had preceded it.

To assess differences in adverse events between the 5 mg and 10 mg SGLT2i groups, safety outcomes were identified, including ketoacidosis, acute kidney injury (AKI), urinary tract infection (including acute pyelonephritis), fall and/or fracture, and other unplanned admission due to SGLT2i use. The operative definition of other unplanned admission due to SGLT2i use was consistent with that used in the previous studies (15). Briefly, it was defined as a hospitalization event occurring during SGLT2i treatment, followed by the absence of a new SGLT2i prescription for at least 3 months after discharge.

To evaluate the consistency of the observed treatment effect, we conducted predefined subgroup analyses according to age (≥70 years), sex, body mass index (BMI) (≥25 kg/m^2^), body weight (<60 kg), fasting glucose (≥126 mg/dL), renal function (eGFR <60 mL/min/1.73m^2^), use of diuretics (loop diuretics and/or spironolactone), CCI (≥3), and concomitant use of ARNI.

### Statistical analysis

Descriptive statistics were presented as means with standard deviations for continuous variables and as counts with percentages for categorical variables. To adjust for baseline differences in covariates related to SGLT2i use, propensity score (PS) matching was performed using a 1:1 pairwise matching strategy, with the 5-mg SGLT2i group serving as the reference. Propensity scores were estimated separately for each dose comparison using logistic regression models that included all covariates described in the Methods section, reflecting differences in baseline characteristics across dose groups. Nearest-neighbor matching without replacement was employed using a caliper width of 0.1 standard deviations of the logit of the PS. Covariate balance after matching was assessed using standardized mean differences (SMDs), with an SMD <0.1 indicating adequate balance.

In the matched cohort, group comparisons were performed using generalized estimating equations to account for within-set correlation induced by PS matching. Incidence rates for the primary outcome were calculated as the number of events divided by total person-time and expressed per 1,000 person-years. Cumulative incidences of the primary and secondary endpoints were illustrated using Kaplan–Meier curves and compared using the log-rank test. Multivariable Cox proportional hazards regression was conducted to estimate the association between SGLT2i dose and the primary outcome, with results reported as HRs and 95% confidence intervals (CIs). The proportional hazards assumption was assessed using Schoenfeld residuals and graphical diagnostics, and no meaningful violations were detected.

Subgroup analyses were performed according to age, sex, BMI, body weight, fasting glucose, eGFR, use of loop diuretics, MRA, CCI and use of ARNI. Outcomes were compared between the 5-mg and 10-mg groups within each subgroup. As a sensitivity analysis, landmark analyses at 6, 9, and 12 months after SGLT2i initiation were conducted by restricting the analysis to patients who were event-free at each landmark time point, to assess the temporal consistency of associations while minimizing the influence of early events, reverse causation, and potential immortal time bias. All statistical analyses were performed using SAS software (version 9.3; SAS Institute, Cary, NC, USA) and R software (version 3.6.3; R Foundation for Statistical Computing, Vienna, Austria). A two-tailed *P* value <0.05 was considered statistically significant.

## Results

### Baseline characteristics of the study participants in total and PS-matched population

A total of 551,760 participants were initially included in this study. The mean participant age was 68.8 ± 13.8 years, and 41.6% were female. The SGLT2i 5 mg and 10 mg groups comprised 2,657 and 52,442 patients, respectively, while 496,661 patients with diabetes and HF did not receive SGLT2i treatment. Compared with the non-SGLT2i group, the SGLT2i group had a higher proportion of females, a greater BMI, and higher body weight. Additionally, the SGLT2i group had a higher prevalence of smoking, a greater number of comorbidities, and a higher prescription rate for cardiovascular medications. In particular, a significant difference in ARNI use was observed between the groups. To account for these baseline differences, PS-matching analysis was performed. In the matched cohort, the SGLT2i 5 mg and 10 mg groups included 2,637 and 13,185 patients, respectively, while the non-SGLT2i group comprised 13,185 patients. The mean age of the PS-matched population was 69.8 years, and 6,673 patients (51.1%) were female. After matching, the SMD for all variables was below 0.1, indicating a well-balanced cohort. The prevalence of HF with reduced ejection fraction based on ARNI prescription was 9.4%, with the non-SGLT2i group exhibiting a numerically higher rate of ARNI use, although this difference was statistically insignificant. The median duration of SGLT2i use was 452.0 (interquartile range, 190.0–932.0) days, with a significantly longer duration observed in the SGLT2i 5 mg group compared with the 10 mg group (516.0 vs. 440.0 days, *P* < .001) in the PS-matched cohort (Table 1). Among the 15,822 SGLT2i users in the matched cohort, 9,349 (59.1%) received dapagliflozin and 6,473 (40.9%) received empagliflozin. Within the 5 mg group, 864 (32.8%) received dapagliflozin and 1,773 (67.2%) received empagliflozin. Within the 10 mg group, 8,485 (64.4%) received dapagliflozin and 4,700 (35.6%) received empagliflozin.

**Table 1:** Baseline characteristics of the propensity-score matched cohort stratified by SGLT2i use.

Characteristic	Non-users (*n* = 13,185)	SGLT2i 10 mg (*n* = 13,185)	SGLT2i 5 mg (*n* = 2,637)	*p*-value	SMD
Age, years	70.4 ± 13.0	69.2 ± 11.8	69.4 ± 11.9	<0.001	0.067
Female sex	6,673 (50.6)	6,825 (51.8)	1,315 (49.9)	0.075	0.025
Income, low 25%	0.26 ± 0.44	0.23 ± 0.42	0.23 ± 0.42	<0.001	0.040
BMI, kg/m^2^	26.1 ± 4.1	26.5 ± 4.2	26.5 ± 4.2	<0.001	0.074
BMI ≥25.0 kg/m^2^	7,759 (58.8)	8,262 (62.7)	1,628 (61.7)	<0.001	0.052
Body weight, kg	67.5 ± 13.9	68.8 ± 13.9	68.5 ± 13.8	<0.001	0.064
Body weight <60 kg	3,959 (30.0)	3,416 (25.9)	711 (27.0)	<0.001	0.061
Alcohol use				<0.001	0.054
Non-drinker	8,168 (61.9)	8,556 (64.9)	1,732 (65.7)		
Mild or moderate drinker	4,386 (33.3)	4,079 (30.9)	791 (30.0)		
Heavy drinker	631 (4.8)	550 (4.2)	114 (4.3)		
Smoke				0.047	0.040
Non-smoker	8,616 (65.3)	8,595 (65.2)	1,750 (66.4)		
Ex-smoker	4,104 (31.1)	4,060 (30.8)	771 (29.2)		
Current smoker	465 (3.5)	530 (4.0)	116 (4.4)		
Underlying disease, *n* (%)					
Hypertension	11,797 (89.5)	11,972 (90.8)	2,402 (91.1)	<0.001	0.036
Dyslipidemia	12,636 (95.8)	12,573 (95.4)	2,511 (95.2)	0.114	0.020
Ischemic heart disease	6,908 (52.4)	7,249 (55.0)	1,433 (54.3)	<0.001	0.035
Atrial fibrillation	3,333 (25.3)	3,328 (25.2)	670 (25.4)	0.984	0.003
Previous stroke	1,559 (11.8)	1,472 (11.2)	297 (11.3)	0.229	0.014
Chronic kidney disease	1,572 (11.9)	1,470 (11.1)	288 (10.9)	0.092	0.021
CCI	5.16 (2.86)	5.05 (2.52)	5.02 (2.54)	0.002	0.035
CCI ≥3	11,063 (83.9)	11,281 (85.6)	2,261 (85.7)	<0.001	0.034
Medication, *n* (%)					
RAS inhibitor	8,550 (64.8)	8,784 (66.6)	1,765 (66.9)	0.005	0.029
Beta-blocker	7,754 (58.8)	7,630 (57.9)	1,525 (57.8)	0.265	0.013
ARNI	1,292 (9.8)	1,216 (9.2)	231 (8.8)	0.126	0.024
MRA	3,309 (25.1)	3,084 (23.4)	602 (22.8)	0.001	0.035
Loop diuretics	6,172 (46.8)	5,832 (44.2)	1,175 (44.6)	<0.001	0.035
Dapagliflozin		8,485 (64.4)	864 (32.8)	<0.001	
Empagliflozin		4,700 (35.6)	1,773 (67.2)	<0.001	
Laboratory findings, *n* (%)					
Hemoglobin, g/dL	13.9 ± 1.7	14.0 ± 1.8	13.9 ± 1.8	<0.001	0.034
Fasting glucose, mg/dL	126.3 ± 47.6	129.3 ± 40.9	128.4 ± 39.7	<0.001	0.047
Estimated GFR, mL/min/1.73m^2^	79.5 ± 20.8	79.1 ± 21.2	78.6 ± 21.5	0.122	0.027
FU duration, year	1.02 [0.31, 2.34]	1.20 [0.51, 2.53]	1.41 [0.55, 2.63]	<0.001	0.112

CCI, Charlson comorbidity index; BMI, body mass index; RAS, renin-angiotensin system; ARNI, angiotensin receptor-neprilysin inhibitor; MRA, mineralocorticoid receptor antagonist; GFR, glomerular filtration rate.

### Study endpoints in the PS-matched cohort

**Figure 2:**
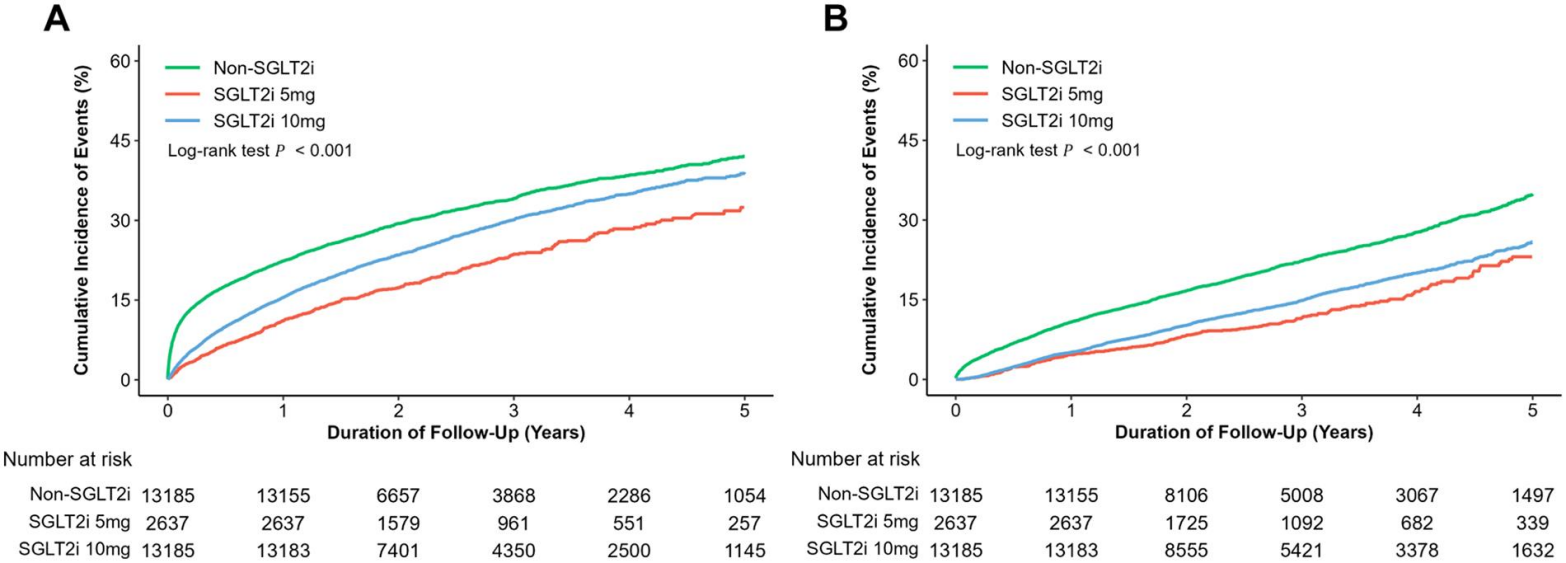
Kaplan–Meier curves for the composite primary outcome **(A)** and all-cause mortality **(B)** All comparisons showed statistically significant differences (log-rank *P* < 0.001), except for the comparison between 5 mg and 10 mg groups in all-cause mortality (log-rank *P* = 0.007).

During the follow-up period, the primary outcome was reached in 6,896 (23.8%) patients or 145.6 per 1,000 person-years. Cardiac death occurred in 738 patients (2.6%), first hospitalization for HF in 6,561 patients (22.6%), and urgent ED visits for HF in 1,353 patients (4.7%). All-cause mortality was observed in 4,035 patients (13.9%). Compared with the non-SGLT2i group, SGLT2i use was associated with significantly lower rates of the composite primary outcome, each of its individual components, and all-cause mortality (Table 2 and Figure 2A). Among SGLT2i users, the incidence of the composite primary outcome was significantly lower in the 5 mg group than in the 10 mg group (16.7% vs. 21.9%, log-rank *P* < 0.001). To compare the risk of the primary outcome between the two dosage groups, Cox regression analysis was performed with the 10 mg group as the reference. The 5 mg group was associated with a significantly lower risk of the composite primary outcome (HR: 0.728, 95% CI: 0.657–0.807, *P* < 0.001), as well as lower risks of hospitalization for HF (HR: 0.726, 95% CI: 0.655–0.806, *P* < 0.001) and all-cause death (HR: 0.85, 95% CI: 0.74–0.97, *P* = 0.019) compared with the 10 mg group (Figure 2B). No significant differences in the incidence of cardiac death or urgent ED visits for HF were observed between the 5 mg and 10 mg SGLT2i groups (Table 2). These significant results for the composite primary outcome remained consistent in a multivariable Cox regression analysis performed in the total population before PS matching. The model was adjusted for demographic, clinical, and laboratory variables used for PS matching (adjusted HR: 0.77, 95% CI: 0.70–0.85, *P* < 0.001).

**Table 2:** Clinical outcome in the propensity score-matched population of patients with diabetes and HF.

Outcome	Non-users	SGLT2i 10 mg	SGLT2i 5 mg	HR (95% CI): 5 mg vs. 10 mg	*p*-value
Composite primary outcome	3,572 (27.1)	2,882 (21.9)	442 (16.8)	0.728 (0.657–0.807)	<0.001
Cardiac death	473 (3.6)	225 (1.7)	41 (1.6)	0.949 (0.678–1.328)	0.760
HF hospitalization	3,348 (25.4)	2,789 (21.2)	424 (16.1)	0.726 (0.655–0.806)	<0.001
Urgent HF-related ED visit	701 (5.3)	556 (4.2)	96 (3.6)	0.853 (0.686–1.061)	0.155
All-cause death	2,317 (17.6)	1,472 (11.2)	246 (9.3)	0.846 (0.735–0.973)	0.019

HF indicates heart failure; HR, hazard ratio; CI, confidence interval; ED, emergency department.

Regarding the composite safety endpoint, events occurred in 317 patients (12.0%) in the 5 mg group and 1,553 patients (11.8%) in the 10 mg group, showing no significant difference between the groups. Most of these events were attributed to other unplanned admissions. The incidence rates of diabetic ketoacidosis, AKI, urinary tract infections including pyelonephritis, and fall-related fractures were all below 1% (Supplementary Table S2).

### Subgroup analysis

**Figure 3:**
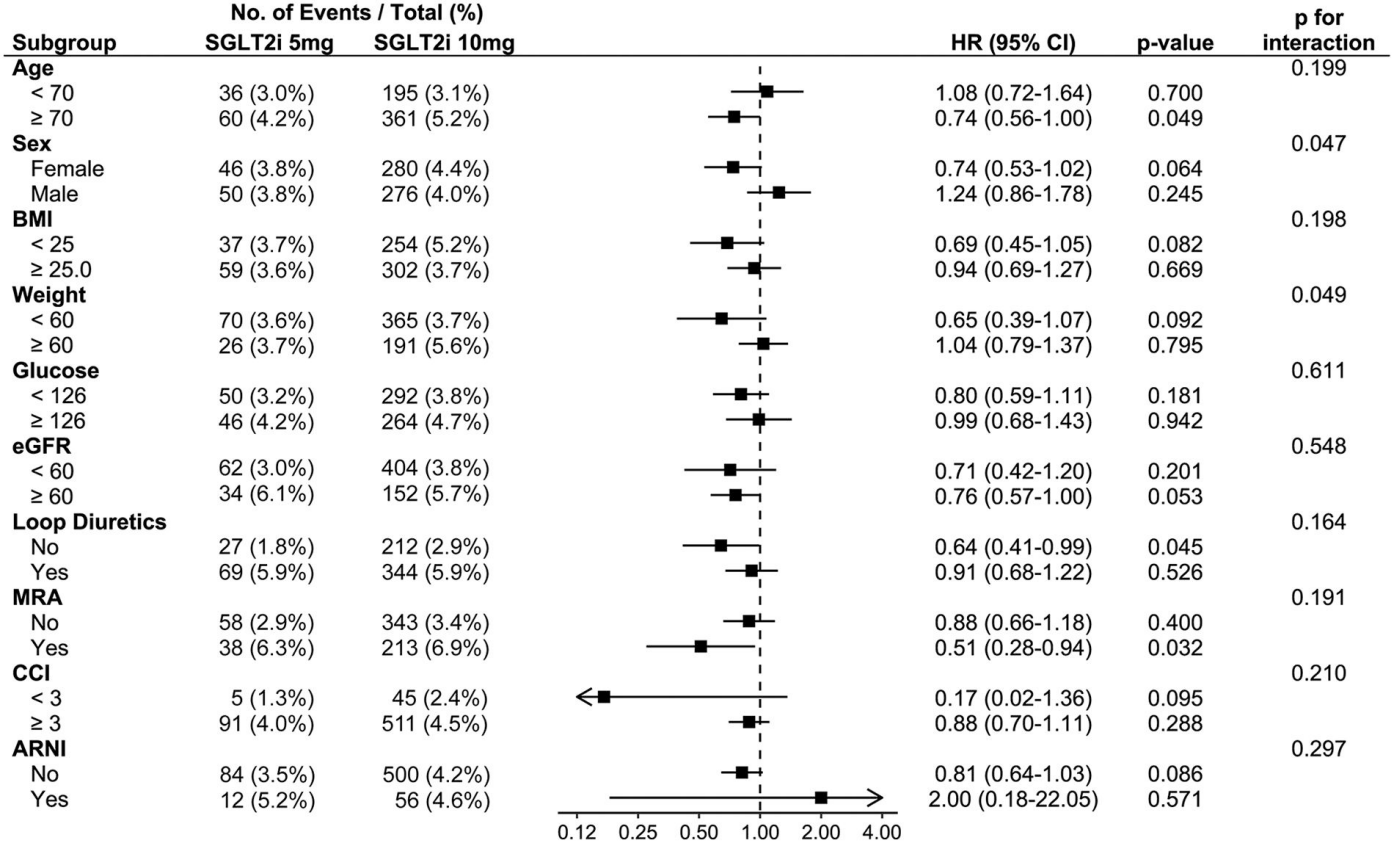
Subgroup analysis of the urgent emergency department visit outcome. ARNI, angiotensin receptor–neprilysin inhibitor; BMI, body mass index; CCI, Charlson comorbidity index; eGFR, estimated glomerular filtration rate; MRA, mineralocorticoid receptor antagonist.

Subgroup analyses were performed based on clinically relevant characteristics, including age, sex, BMI, body weight, fasting glucose level, renal function, diuretic use, and comorbidity burden. A significant interaction was observed for the composite primary outcome according to ARNI use (*P*-for-interaction = 0.039) (Supplementary Table S3). Among patients not receiving ARNI, those in the SGLT2i 5 mg group were associated with a significantly lower risk of the composite endpoint compared with those in the SGLT2i 10 mg group (HR: 0.69; 95% CI: 0.61–0.77; *P* < 0.001), whereas no significant difference was observed among ARNI users (HR: 0.80; 95% CI: 0.48–1.32; *P* = 0.375). For hospitalization due to HF, a similar interaction by ARNI use was found (*P*-for-interaction = 0.018), with a lower hazard associated with SGLT2i 5 mg observed predominantly in the non-ARNI group (HR: 0.68; 95% CI: 0.61–0.77; *P* < 0.001). Regarding urgent ED visits due to HF, a significant interaction by sex was detected (*P*-for-interaction = 0.047), with women showing a numerically greater risk reduction trend in the SGLT2i 5 mg group (HR: 0.74; 95% CI: 0.53–1.02) than that in men, although this difference did not reach statistical significance. An interaction by body weight (cutoff at 60 kg) was also found for the urgent ED visits (*P*-for-interaction = 0.049). The SGLT2i 5 mg group was associated with a numerically lower hazard in those weighing <60 kg (HR: 0.65; 95% CI: 0.39–1.07), whereas no such association was observed in patients weighing ≥60 kg (HR: 1.04; 95% CI: 0.79–1.37); however, these differences were not statistically significant in either weight group. Regarding age, although the test for interaction was not statistically significant (*P*-for-interaction = 0.199), the SGLT2i 5 mg dose was associated with a significantly lower risk of urgent ED visits in patients aged ≥70 years (HR: 0.74, 95% CI: 0.56–1.00, *P* = 0.049), whereas no such association was observed in those aged <70 years (HR: 1.08, 95% CI: 0.72–1.64, *P* = 0.700) (Figure 3). Among safety outcomes, AKI showed significant interactions with both BMI and body weight (Supplementary Table S4). When stratified by BMI, a significant interaction was observed (*P*-for-interaction = 0.006). In patients with BMI ≥25 kg/m^2^, the SGLT2i 5 mg group was associated with a higher risk of AKI compared with the 10 mg group (HR: 2.54; 95% CI: 1.15–5.60; *P* = 0.021). In contrast, although the difference did not reach statistical significance, the 5 mg group was associated with a numerically lower risk of AKI in patients with BMI <25 kg/m^2^ (HR: 0.27; 95% CI: 0.06–1.20; *P* = 0.086). A significant interaction was also noted when stratified by body weight using 60 kg as the cutoff (*P*-for-interaction = 0.034). Although neither subgroup reached statistical significance individually, the HR was numerically lower in patients weighing <60 kg (HR: 0.31; 95% CI: 0.04–2.59; *P* = 0.282), and numerically higher in those weighing ≥60 kg (HR: 1.97; 95% CI: 0.98–3.96; *P* = 0.059).

### Sensitivity analysis by follow-up duration

To assess the robustness of the observed association and to minimize the influence of early events, we conducted predefined landmark analyses by restricting the analysis to patients who remained free of events prior to specific time points. In the PS-matched cohort, separate analyses were performed among patients who were event-free at 6, 9, and 12 months after initiation of SGLT2i therapy (Supplementary Table S5). Across all landmark time points, the incidence of the composite primary outcome consistently tended to be lower in the 5-mg group compared with the 10-mg group. Specifically, the hazard ratio for the 5-mg group vs. the 10-mg group was 0.79 (95% CI: 0.69–0.90; *P* < 0.001) at 6 months, 0.82 (95% CI: 0.71–0.94; *P* = 0.006) at 9 months, and 0.80 (95% CI: 0.68–0.94; *P* = 0.006) at 12 months, demonstrating consistent associations across landmark time points.

## Discussion

In this large-scale nationwide cohort study, we evaluated the clinical effectiveness of low-dose SGLT2i in patients with diabetes and HF. Consistent with findings from previous randomized controlled trials, SGLT2i use was associated with a lower incidence of the composite primary outcome—comprising cardiac death, HF hospitalization and urgent ED visits for HF—compared with SGLT2i non-use. Notably, the 5 mg SGLT2i group was associated with comparable risk to the 10 mg group for the composite primary outcome—a finding primarily driven by reduced HF-related hospitalizations—as well as for all-cause mortality. Subgroup analyses revealed significant interactions by sex and body weight in components of the study endpoint. These findings highlight the potential clinical utility of a lower SGLT2i dose in patients with HF, warranting further investigation into its efficacy and safety in real-world practice.

Rosiglitazone is an oral antihyperglycemic agent of the thiazolidinedione class approved by the U.S. Food and Drug Administration (FDA) in 1999. However, a 2007 meta-analysis reported that rosiglitazone may increase the risk of myocardial infarction and cardiovascular death (21). This finding led to subsequent regulatory restrictions on its use. Subsequently, the FDA issued new guidelines mandating that all new antidiabetic therapies demonstrate cardiovascular safety through large-scale randomized controlled trials, requiring evidence that they do not increase the risk of major adverse cardiovascular events. Since the first phase 3 trial of empagliflozin in 2015, SGLT2i have been evaluated in large multicenter trials in patients with HF regardless of diabetes, based on their ability to reduce HF hospitalization (1, 4–6, 13). However, to expedite the demonstration of clinical benefit, these trials were initiated without prior phase 2 studies specifically designed to identify the optimal dose in patients with HF. Consequently, the optimal dose of SGLT2i that maximizes efficacy while minimizing adverse effects in patients with HF remains unclear, and current clinical guidelines recommend only a single fixed dose. However, unlike participants in large phase 3 clinical trials, real-world studies have reported suboptimal adherence to SGLT2i in routine clinical practice (14, 15). This suboptimal adherence is likely attributable to various adverse effects experienced during treatment. However, no study has investigated dose adjustment strategies to address this issue. The present study provides foundational real-world evidence from a nationwide observational cohort that may inform the design of future dose-optimization trials.

The factors contributing to poor adherence to SGLT2i remain unclear. However, previous observational studies based on nationwide claims data have identified older age, concomitant use of diuretics, and lower BMI or weight as risk factors for treatment discontinuation (15). In line with recent studies showing that the use of SGLT2i in older patients with HF is associated with favorable cardiovascular outcomes, the lower-dose association in our cohort was consistent across age strata (22). In our subgroup analysis, a significant interaction was observed between participants weighing <60 kg and those weighing ≥60 kg with respect to urgent ED visits due to HF. For safety outcomes, a statistically significant interaction by BMI was observed for AKI, with a numerically higher AKI signal in the 5 mg group among patients with BMI ≥25 kg/m^2^. However, this finding lacks biological plausibility—SGLT2i renoprotection is mediated through tubuloglomerular feedback and reductions in intraglomerular pressure, neither of which has any established interaction with BMI, and the early eGFR decline observed with SGLT2i is hemodynamic and reversible rather than true kidney injury. Given the small absolute number of AKI events (*n* = 75 across both dose groups), this signal most likely reflects residual confounding, multiple-testing artifact, or random variation, and should be regarded as exploratory and hypothesis-generating only. These findings collectively suggest that prospective or randomized controlled trials should be considered to evaluate the potential indication for low-dose SGLT2i in patients with female sex or body weight <60 kg.

Our findings should be interpreted within the context of existing observational evidence on low-dose SGLT2i. The DAPPER study suggested that low-dose dapagliflozin may reduce cardiovascular events in diabetic HF (23). Also, Braver et al. reported broadly comparable outcomes between low- and standard-dose SGLT2i in a real-world retrospective cohort, although patients receiving lower doses were older with higher comorbidity burden (24). With respect to renal safety, dedicated kidney sub-analyses of EMPEROR-Reduced AKI and EMPEROR-Preserved kidney sub-analysis (25, 26), as well as meta-analyses of SGLT2i trials (27), have consistently shown that SGLT2i are associated with reduced rather than increased AKI risk and with preservation of long-term kidney function, further supporting the implausibility of a dose-related increase in AKI risk in our cohort. The present study extends these observations in a substantially larger nationwide cohort with prespecified agent stratification, while sharing the inherent limitations of observational data—residual confounding, selection bias, and lack of randomization. Our findings are therefore hypothesis-generating and cannot replace evidence from randomized dose-finding trials, in which the standard 10 mg dose remains the guideline-recommended reference.

This study is the first to report the cardiovascular benefits of low-dose SGLT2i in patients with diabetes and HF using a nationwide, large-scale claims database. It also highlights the need to determine the optimal dosing of SGLT2i dose for HF, given that approval for this indication was granted without preceding phase II trials. Our findings underscore the potential impact of appropriate SGLT2i dosing on improving clinical outcomes in patients with HF.

### Study limitations

This study has several limitations. First, during the study period, SGLT2i in Korea were reimbursed solely for the treatment of diabetes, not HF. As a result, we could not assess the outcomes of low-dose SGLT2i among HF patients without diabetes. Nevertheless, considering the low incidence of DKA within our primary outcomes and the consistent efficacy of SGLT2i in both patients with and without diabetes reported in prior studies, we believe our findings may be generalizable to the broader population of patients with HF (1, 5, 6, 13). Second, although PS matching balanced measured covariates, dose selection was not randomized; clinicians may preferentially prescribe a lower dose to frailer or lower-weight patients, or conversely to those with milder disease. In addition, the NHIS database does not capture frailty indices, NYHA class, or prescriber preferences. Residual confounding by indication may therefore have biased the observed associations in either direction, and our findings should be interpreted as associative rather than causal. Third, NHIS claims data do not contain HbA1c, LVEF, NT-proBNP, or detailed echocardiographic measurements. Accordingly, we could not differentiate HF with reduced EF (HFrEF) from mildly reduced EF and preserved EF, evaluate diastolic function, or assess longitudinal LVEF response to therapy. As a partial surrogate for HFrEF, we used ARNI prescription status, since ARNI reimbursement in Korea is restricted to patients with HFrEF. Interestingly, the dose-related associations differed according to ARNI use, suggesting potential phenotype-dependent effects. Nevertheless, interpretation of dose effects across specific HF phenotypes requires confirmation in studies incorporating detailed echocardiographic and biomarker data. Fourth, only dapagliflozin and empagliflozin were included in this study, and analyses according to individual SGLT2i agent were not performed. Because different SGLT2i may have distinct pharmacokinetic profiles and dose-response characteristics, potential heterogeneity in dose-related effects between agents could not be evaluated. Further studies specifically designed to compare dose effects according to individual SGLT2i type are warranted. Lastly, as our study population consisted exclusively of Korean individuals, further research is needed to determine whether these results can be generalized to other ethnic populations.

## Conclusion

This nationwide cohort study suggests that a 5 mg dose of SGLT2i is associated with comparable risk to 10 mg for the composite primary outcome and all-cause mortality. Whether a low-dose SGLT2i may serve as an alternative in selected patient groups warrants further evaluation in dedicated dose-finding trials.

## Data Availability

The datasets analyzed in this study are not publicly available because they were obtained from the Korean National Health Insurance Service (NHIS) under a data use agreement. NHIS claims data are owned by the NHIS and cannot be redistributed by individual researchers. Qualified researchers may apply for access directly through the NHIS Data Sharing Service (National Health Insurance Sharing Service; https://nhiss.nhis.or.kr/) in accordance with NHIS policy and applicable Korean law. Further inquiries regarding the dataset used in this study can be directed to the corresponding author. Requests to access these datasets should be directed to the Korean National Health Insurance Sharing Service (https://nhiss.nhis.or.kr/).
