# Supplementary material for: Effectiveness of low-dose SGLT2 inhibitors in diabetic patients with heart failure: a nationwide cohort study
Source: Front Cardiovasc Med. 2026 Jun 22;13:1859003. doi: 10.3389/fcvm.2026.1859003 (PMC13333392; doi:10.3389/fcvm.2026.1859003)
Supplement: Supplementary file 1 [file Table1.docx]

**Supplementary Table 1. Definitions of covariates.**

| **Diagnosis** | **ICD-10-CM code and definition** | **Diagnostic definition** |
| --- | --- | --- |
| **Hypertension** | I10-I13, I15 | Admission or outpatient department≥3 |
| **Dyslipidemia** | E78 | Admission≥1 or outpatient department≥3 |
| **Ischemic heart disease** | I20-25 | Admission or outpatient clinic≥2 |
| **Heart failure** | I50 | Admission or outpatient clinic≥1 |
| **Atrial fibrillation** | I48 | Admission or outpatient clinic≥2 |
| **Previous stroke** | I63, I64, I67.8-9 | Admission or outpatient clinic≥2 |
| **Chronic kidney disease** | N18 | Admission or outpatient clinic≥2 |

**Supplementary Table 2.** **Safety outcome in the propensity score-matched population of diabetic patients with HF.**

| **Outcome** | **SGLT2i 10mg** | | **SGLT2i 5mg** | | **5mg vs 10mg** | |
| --- | --- | --- | --- | --- | --- | --- |
|  | **Events (%)** | **Incidence rate (per 1,000)** | **Events (%)** | **Incidence rate (per 1,000)** | **Hazard Ratio (95% CI)** | **p-vaule** |
| **Safety outcome** | 1553 (11.78) | 61.75 | 317 (12.02) | 63.70 | 1.009 (0.890-1.144) | 0.884 |
| Ketoacidosis | 0 (0.00) | 0.00 | 1 (0.04) | 0.18 | - | - |
| Acute kidney injury | 58 (0.44) | 2.10 | 17 (0.64) | 3.11 | 1.304 (0.736-2.312) | 0.364 |
| Urinary tract infection, including pyelonephritis | 37 (0.28) | 1.34 | 4 (0.15) | 0.73 | 0.527 (0.185-1.498) | 0.229 |
| Slipdown and/or fracture | 37 (0.28) | 1.34 | 10 (0.38) | 1.82 | 1.246 (0.592-2.623) | 0.562 |
| Other unplanned admission | 1425 (10.81) | 56.22 | 288 (10.92) | 57.22 | 1.002 (0.879-1.142) | 0.979 |

**Supplementary Table 3. Subgroup analysis for clinical outcomes**

|  | Composite primary endpoint | | | Cardiac death | | | HF hospitalization | | | Urgent HF related ED visit | | |
| --- | --- | --- | --- | --- | --- | --- | --- | --- | --- | --- | --- | --- |
|  | Hazard Ratio (95% CI) | p-vaule | p for interaction | Hazard Ratio (95% CI) | p-vaule | p for interaction | Hazard Ratio (95% CI) | p-vaule | p for interaction | Hazard Ratio (95% CI) | p-vaule | p for interaction |
| Age |  |  | 0.424 |  |  | 0.657 |  |  | 0.333 |  |  | 0.199 |
| < 70 | 0.770 (0.634-0.935) | 0.008 |  | 0.825 (0.372-1.826) | 0.635 |  | 0.786 (0.646-0.957) | 0.016 |  | 1.084 (0.719-1.636) | 0.700 |  |
| ≥ 70 | 0.690 (0.599-0.794) | <0.001 |  | 0.919 (0.604-1.398) | 0.693 |  | 0.687 (0.596-0.792) | <0.001 |  | 0.745 (0.555-0.999) | 0.049 |  |
| Sex |  |  | 0.113 |  |  | 0.362 |  |  | 0.101 |  |  | 0.047 |
| Female | 0.663 (0.565-0.778) | <0.001 |  | 0.851 (0.493-1.469) | 0.563 |  | 0.653 (0.555-0.769) | <0.001 |  | 0.736 (0.531-1.018) | 0.064 |  |
| Male | 0.796 (0.674-0.941) | 0.007 |  | 1.236 (0.711-2.149) | 0.453 |  | 0.792 (0.669-0.938) | 0.007 |  | 1.239 (0.863-1.780) | 0.245 |  |
| Body mass index, kg/m2 |  |  | 0.579 |  |  | 0.913 |  |  | 0.639 |  |  | 0.198 |
| < 25 | 0.686 (0.565-0.834) | <0.001 |  | 0.765 (0.439-1.334) | 0.346 |  | 0.702 (0.577-0.855) | <0.001 |  | 0.689 (0.452-1.048) | 0.082 |  |
| ≥ 25.0 | 0.668 (0.575-0.777) | <0.001 |  | 0.866 (0.498-1.506) | 0.611 |  | 0.669 (0.575-0.780) | <0.001 |  | 0.936 (0.690-1.269) | 0.669 |  |
| Body weight, kg |  |  | 0.871 |  |  | 0.438 |  |  | 1.000 |  |  | 0.049 |
| ≥ 60 | 0.730 (0.638-0.834) | <0.001 |  | 1.148 (0.721-1.828) | 0.560 |  | 0.732 (0.639-0.838) | <0.001 |  | 1.038 (0.786-1.371) | 0.795 |  |
| < 60 | 0.775 (0.615-0.977) | 0.031 |  | 0.907 (0.462-1.783) | 0.777 |  | 0.795 (0.630-1.003) | 0.053 |  | 0.646 (0.389-1.074) | 0.092 |  |
| Fasting glucose, mg/dL |  |  | 0.195 |  |  | 0.112 |  |  | 0.089 |  |  | 0.611 |
| < 126 | 0.732 (0.634-0.845) | <0.001 |  | 0.752 (0.449-1.259) | 0.278 |  | 0.744 (0.643-0.860) | <0.001 |  | 0.805 (0.586-1.106) | 0.181 |  |
| ≥ 126 | 0.662 (0.547-0.801) | <0.001 |  | 1.197 (0.671-2.135) | 0.543 |  | 0.649 (0.535-0.788) | <0.001 |  | 0.986 (0.680-1.431) | 0.942 |  |
| Estimated GFR, mL/min/1.73m2 |  |  | 0.270 |  |  | 0.759 |  |  | 0.306 |  |  | 0.548 |
| ≥ 60 | 0.664 (0.583-0.756) | <0.001 |  | 0.855 (0.550-1.332) | 0.489 |  | 0.668 (0.585-0.762) | <0.001 |  | 0.757 (0.571-1.003) | 0.053 |  |
| < 60 | 0.674 (0.514-0.885) | 0.004 |  | 0.696 (0.305-1.587) | 0.389 |  | 0.673 (0.512-0.884) | 0.004 |  | 0.712 (0.423-1.198) | 0.201 |  |
| Loop diuretics |  |  | 0.713 |  |  | 0.729 |  |  | 0.807 |  |  | 0.164 |
| No | 0.674 (0.556-0.816) | <0.001 |  | 1.080 (0.468-2.493) | 0.857 |  | 0.673 (0.555-0.818) | <0.001 |  | 0.641 (0.415-0.990) | 0.045 |  |
| Yes | 0.722 (0.621-0.838) | <0.001 |  | 1.011 (0.657-1.558) | 0.959 |  | 0.715 (0.615-0.832) | <0.001 |  | 0.908 (0.675-1.222) | 0.526 |  |
| MRA |  |  | 0.968 |  |  | 0.802 |  |  | 0.697 |  |  | 0.191 |
| No | 0.698 (0.607-0.804) | <0.001 |  | 1.033 (0.619-1.722) | 0.902 |  | 0.706 (0.612-0.814) | <0.001 |  | 0.882 (0.659-1.181) | 0.400 |  |
| Yes | 0.665 (0.516-0.857) | 0.002 |  | 0.863 (0.374-1.996) | 0.731 |  | 0.663 (0.514-0.855) | 0.002 |  | 0.510 (0.275-0.944) | 0.032 |  |
| CCI |  |  | 0.897 |  |  | 0.899 |  |  | 0.787 |  |  | 0.210 |
| < 3 | 0.390 (0.197-0.773) | 0.007 |  | 1.000 (0.063-15.988) | 1.000 |  | 0.410 (0.206-0.816) | 0.011 |  | 0.170 (0.021-1.362) | 0.095 |  |
| ≥ 3 | 0.708 (0.634-0.792) | <0.001 |  | 0.935 (0.655-1.333) | 0.709 |  | 0.708 (0.633-0.793) | <0.001 |  | 0.883 (0.701-1.111) | 0.288 |  |
| ARNI |  |  | 0.039 |  |  | 0.619 |  |  | 0.018 |  |  | 0.297 |
| No | 0.686 (0.611-0.770) | <0.001 |  | 0.996 (0.680-1.458) | 0.982 |  | 0.681 (0.606-0.766) | <0.001 |  | 0.814 (0.644-1.029) | 0.086 |  |
| Yes | 0.795 (0.479-1.320) | 0.375 |  | 2.562 (0.225-29.121) | 0.448 |  | 0.850 (0.513-1.406) | 0.526 |  | 2.000 (0.181-22.056) | 0.571 |  |

GFR indicates glomerular filtration rate; MRA, mineralocorticoid receptor antagonist; CCI, Charlson comorbidity index; ARNI

**Supplementary Table 4. Subgroup analysis for safety outcomes**

|  | **Acute kidney injury** | | | **Urinary tract infection,  including pyelonephritis** | | | **Slipdown and/or fracture** | | | **Other unplanned admission** | | |
| --- | --- | --- | --- | --- | --- | --- | --- | --- | --- | --- | --- | --- |
|  | **Hazard Ratio (95% CI)** | **p-vaule** | **p for interaction** | **Hazard Ratio (95% CI)** | **p-vaule** | **p for interaction** | **Hazard Ratio (95% CI)** | **p-vaule** | **p for interaction** | **Hazard Ratio (95% CI)** | **p-vaule** | **p for interaction** |
| **Age** |  |  | 0.751 |  |  | 0.976 |  |  | 0.459 |  |  | 0.969 |
| < 70 | 1.483 (0.434-5.075) | 0.530 |  | 0.888 (0.083-9.537) | 0.922 |  | 0.544 (0.116-2.554) | 0.440 |  | 1.029 (0.822-1.289) | 0.801 |  |
| ≥ 70 | 1.136 (0.552-2.336) | 0.729 |  | 0.659 (0.186-2.334) | 0.519 |  | 1.212 (0.485-3.024) | 0.681 |  | 0.997 (0.827-1.202) | 0.975 |  |
| **Sex** |  |  | 0.869 |  |  | 0.814 |  |  | 0.551 |  |  | 0.487 |
| Female | 1.543 (0.689-3.452) | 0.292 |  | 0.516 (0.148-1.794) | 0.298 |  | 1.174 (0.364-3.780) | 0.789 |  | 1.021 (0.842-1.238) | 0.834 |  |
| Male | 1.041 (0.377-2.874) | 0.938 |  | 0.580 (0.055-6.102) | 0.650 |  | 1.515 (0.497-4.622) | 0.465 |  | 0.945 (0.755-1.184) | 0.624 |  |
| **Body mass index, kg/m2** |  |  | 0.006 |  |  | - |  |  | 0.219 |  |  | 0.182 |
| < 25 | 0.267 (0.059-1.204) | 0.086 |  | - | - |  | 1.043 (0.293-3.709) | 0.948 |  | 1.026 (0.803-1.311) | 0.839 |  |
| ≥ 25.0 | 2.538 (1.151-5.597) | 0.021 |  | 0.448 (0.100-2.008) | 0.294 |  | 0.643 (0.179-2.303) | 0.497 |  | 0.906 (0.747-1.098) | 0.313 |  |
| **Body weight, kg** |  |  | 0.034 |  |  | - | - |  | 0.402 |  |  | 0.171 |
| ≥ 60 | 1.965 (0.975-3.960) | 0.059 |  | 0.419 (0.094-1.863) | 0.253 |  | 0.861 (0.316-2.341) | 0.769 |  | 0.936 (0.788-1.112) | 0.450 |  |
| < 60 | 0.314 (0.038-2.586) | 0.282 |  | - | - |  | 1.068 (0.189-6.020) | 0.941 |  | 1.089 (0.800-1.483) | 0.587 |  |
| **Fasting glucose, mg/dL** |  |  | 0.858 |  |  | 0.695 |  |  | 0.450 |  |  | 0.776 |
| < 126 | 1.329 (0.598-2.953) | 0.485 |  | 0.508 (0.114-2.256) | 0.373 |  | 1.071 (0.372-3.085) | 0.898 |  | 0.996 (0.819-1.211) | 0.964 |  |
| ≥ 126 | 0.804 (0.248-2.604) | 0.716 |  | 0.161 (0.020-1.273) | 0.083 |  | 2.656 (0.635-11.108) | 0.181 |  | 1.008 (0.807-1.258) | 0.944 |  |
| **Estimated GFR, mL/min/1.73m2** |  |  | 0.230 |  |  | - |  |  | 0.592 |  |  | 0.116 |
| ≥ 60 | 0.878 (0.347-2.223) | 0.783 |  | 0.712 (0.236-2.147) | 0.546 |  | 1.143 (0.482-2.711) | 0.761 |  | 1.045 (0.894-1.222) | 0.582 |  |
| < 60 | 3.816 (0.895-16.267) | 0.070 |  | - | - |  | 0.879 (0.075-10.261) | 0.918 |  | 0.745 (0.510-1.088) | 0.128 |  |
| **Loop diuretics** |  |  | 0.827 |  |  | 0.936 |  |  | 0.698 |  |  | 0.389 |
| No | 2.758 (0.657-11.573) | 0.166 |  | 0.623 (0.130-2.973) | 0.553 |  | 1.778 (0.570-5.552) | 0.322 |  | 1.007 (0.822-1.234) | 0.945 |  |
| Yes | 1.414 (0.641-3.120) | 0.390 |  | 0.536 (0.112-2.574) | 0.436 |  | 0.967 (0.240-3.898) | 0.962 |  | 0.865 (0.690-1.084) | 0.207 |  |
| **MRA** |  |  | 0.686 |  |  | 0.888 |  |  | 0.652 |  |  | 0.561 |
| No | 1.599 (0.718-3.563) | 0.251 |  | 0.516 (0.152-1.755) | 0.290 |  | 1.430 (0.590-3.463) | 0.428 |  | 1.027 (0.879-1.199) | 0.741 |  |
| Yes | 1.000 (0.141-7.099) | 1.000 |  | 1.732 (0.098-30.763) | 0.708 |  | 1.414 (0.193-10.340) | 0.733 |  | 1.074 (0.701-1.646) | 0.742 |  |
| **CCI** |  |  | - |  |  | - |  |  | - |  |  | 0.790 |
| < 3 | - | - |  | - | - |  | - | - |  | 0.711 (0.345-1.466) | 0.355 |  |
| ≥ 3 | 1.281 (0.704-2.331) | 0.417 |  | 0.580 (0.199-1.688) | 0.317 |  | 1.120 (0.505-2.481) | 0.780 |  | 1.002 (0.869-1.154) | 0.983 |  |
| **ARNI** |  |  | - |  |  | - |  |  | 0.111 |  |  | 0.461 |
| No | 1.333 (0.709-2.507) | 0.372 |  | 0.533 (0.187-1.522) | 0.240 |  | 1.447 (0.677-3.095) | 0.340 |  | 1.023 (0.893-1.172) | 0.746 |  |
| Yes | - | - |  | - | - |  | 0.000 (0.000-Inf) | 1.000 |  | 1.208 (0.398-3.669) | 0.739 |  |

CCI, Charlson comorbidity index; RAS, renin-angiotensin system; ARNI, angiotensin receptor-neprilysin inhibitor; MRA, mineralocorticoid receptor antagonist; GFR, glomerular filtration rate.

**Supplementary Table 5. Sensitivity analysis for the primary composite endpoint according to different subgroups of patients who remained free of events up to 6, 9, and 12 months after SGLT2i initiation.**

|  | **SGLT2i 10mg** | **SGLT2i 5mg** | **HR (95% CI): 5mg vs. 10mg** | **p-value** |
| --- | --- | --- | --- | --- |
| 6 months | 1653 (16.48) | 282 (13.79) | 0.787 (0.690-0.897) | <0.001 |
| 9 months | 1353 (15.71) | 242 (13.42) | 0.817 (0.708-0.943) | 0.006 |
| 12 months | 1105 (14.80) | 194 (12.19) | 0.799 (0.680-0.937) | 0.006 |

HR, hazard ratio; CI, confidence interval
